# Occupational medicine: strategies to promote health and well-being in the workplace

**DOI:** 10.47626/1679-4435-2025-1435

**Published:** 2025-09-23

**Authors:** Jordana Carneiro Rodrigues da Cunha, Amanda Caroline de Freitas Barbosa, Isadora de Bessa Guimarães, Samara Gomes Costa, Raul Vidica Teodoro Barcelos, Joana Darc Silvério Porto

**Affiliations:** 1 Medicina, Universidade de Rio Verde, Rio Verde, GO, Brazil; 2 Medicina, Pontifícia Universidade Católica de Goiás, Goiânia, GO, Brazil; 3 Ciências da Saúde, Universidade Federal de Goiás, Goiânia, GO, Brazil

**Keywords:** occupational medicine, occupational health, quality of life, health promotion, medicina do trabalho, saúde ocupacional, qualidade de vida, promoção da saúde

## Abstract

This study analyzed workplace health and well-being promotion strategies through a narrative literature review, focusing on national and international approaches implemented between 2015 and 2024. The results highlighted that integrated actions, combining medical surveillance and psychosocial support, reduce occupational risks, increase productivity, and strengthen employee-company bonds. Models such as Workplace Health Promotion Embedded in Medical Surveillance and Total Worker Health^®^ stood out for promoting physical and mental health, while Hong Kong’s Joyful@Healthy Workplace program demonstrated the effectiveness of combining educational campaigns and ergonomically adapted environments. The success of these strategies depends on leadership commitment, active employee participation, and the development of an organizational culture focused on well-being, adapted to each context’s socio-cultural and economic specificities. In Brazil, implementing these practices requires investments in occupational health, public-private partnerships, and developing indicators to measure their long-term impacts. It is concluded that adopting holistic and continuous approaches is essential to create healthier, more inclusive, and productive workplaces, benefiting both individuals and organizations.

## Abstract (pt)

Este estudo analisou, por meio de uma revisão narrativa da literatura, estratégias de promoção da saúde e do bem-estar no ambiente laboral, com foco em abordagens nacionais e internacionais implementadas entre 2019 e 2024. Os resultados evidenciaram que ações integradas, associadas à vigilância médica e ao suporte psicossocial, reduzem riscos ocupacionais, aumentam a produtividade e fortalecem o vínculo entre colaboradores e empresas. Modelos como o Workplace Health Promotion Embedded in Medical Surveillance e o Total Worker Health^®^ destacaram-se por promover a saúde física e mental, enquanto o programa Joyful@Healthy Workplace, de Hong Kong, demonstrou a eficácia da combinação entre campanhas educativas e ambientes ergonomicamente adequados. O sucesso dessas estratégias depende do comprometimento das lideranças, da participação ativa dos colaboradores e da construção de uma cultura organizacional voltada ao bem-estar, adaptada às particularidades socioculturais e econômicas de cada contexto. No Brasil, a implementação dessas práticas exige investimentos em saúde ocupacional e parcerias público-privadas, além do desenvolvimento de indicadores capazes de mensurar seus impactos a longo prazo. Conclui-se que a adoção de abordagens holísticas e contínuas é essencial para criar ambientes de trabalho mais saudáveis, inclusivos e produtivos, beneficiando tanto os indivíduos quanto as organizações.

## INTRODUCTION

In the work environment, which involves complex dynamics that interconnect physical, psychological and social factors, working conditions and interpersonal interactions directly affect employee health, transcending the occupational sphere and affecting quality of life and professional performance.^1,2^ Because intensifying production demands and technological transformations have increased the incidence of work-related disorders, strategies are needed to strengthen worker well-being, which is a harmony of physical health, emotional balance, and job satisfaction.^3,4^

In this context, occupational medicine, an interdisciplinary field that combines knowledge from public health, organizational psychology, and people management, can develop initiatives to mitigate the negative effects of work activities while fostering healthier and more productive work environments.^5^ The effectiveness of such practices depends on an organizational culture that recognizes employee health as a strategic element of business performance, consolidating policies and processes that integrate health surveillance, the promotion of well-being, and psychosocial support, considering the characteristics of the economic sector and individual workers.^6,7^

The complexity of this scenario is associated with the multifaceted nature of health-related factors in the workplace, ranging from ergonomic conditions and environmental risks to subjective aspects, such as stress, motivation, and professional recognition.^8,9^ A lack of effective health policies can compromise worker health, leading to absenteeism and lost productivity, while also affecting organizational sustainability, resulting in increased health care costs and cases of temporary or permanent disability.^5,10^ Given this scenario, it is essential to investigate how health and well-being promotion strategies can be structured into the daily routine of companies, transcending isolated initiatives and consolidating practices that favor both individual development and institutional growth.^2,4^

This study’s relevance is supported by the role played by occupational health in building more just and balanced societies, given that work is an essential dimension of personal fulfillment and social inclusion.^6^ Understanding different health and well-being promotion strategies in national and international contexts allows us to identify best practices that can be adapted to the Brazilian context, contributing to more effective public policies and improved corporate health programs.^1,9^ Analyzing the effects of such interventions on indicators like absenteeism, presenteeism, staff turnover, and job satisfaction can facilitate decision-making in the business environment and the development of government initiatives to improve worker health.^3,5^

We hypothesized that integrated strategies combining health surveillance with occupational illness prevention and well-being promotion programs can reduce occupational risk, increase productivity, and improve organizational climate, benefitting both workers and companies.^4,9^ However, for such initiatives to achieve their objectives, several challenges must be overcome, including cultural resistance, a lack of investment in occupational health, and the difficulty of measuring long-term results, which requires the commitment of leaders and the active participation of employees at all stages of the process.^1,9^

Thus, this review investigated the most effective strategies reported in the literature for promoting health and well-being in the workplace, considering the different factors that influence worker quality of life and the challenges companies face in implementing them. The overall objective was to analyze the main strategies used in different organizational contexts, identifying elements that contribute to their success and the obstacles that limit their effectiveness. Specifically, we aimed to: (i) examine strategies to promote physical and mental health in the workplace, highlighting their effects on employee productivity and well-being; (ii) identify the organizational and cultural factors that influence adherence to and the effectiveness of these actions; and (iii) analyze best practices in international contexts, assessing their applicability and potential for Brazilian companies, with a view to improving national occupational health policies.

## METHODS

The objective of this narrative review was to gather, analyze, and synthesize the scientific knowledge on strategies in occupational medicine to promote health and well-being in the workplace in national and international contexts.

The study search and selection process were conducted in recognized databases, including PubMed Central, Scopus, LILACS, Latindex, EBSCO, and BMC Public Health to ensure the representativeness and quality of the evidence. The search terms were defined using BIREME’s Health Sciences Descriptors and the U.S. National Library of Medicine’s Medical Subject Headings and combined as follows: “occupational medicine” AND “occupational health” AND “well-being at work”; “health promotion” AND “workplace environment” AND “quality of work life.” We sought to include studies that addressed both interventions aimed at preventing health problems and those aimed at strengthening the physical, mental, and social well-being of workers. The publication time frame was limited to 2019-2024 due to the relevance of recent trends and challenges in occupational health.

Original articles, systematic reviews, experience reports, and observational studies published in Portuguese, English, or Spanish were eligible, provided they presented a clearly described methodology and results that demonstrated the strategy’s effects of on health and productivity indicators. Publications that did not specify the organizational context of the interventions were excluded, as were those whose approach was restricted to technical aspects of occupational medicine, without considering the interactions between health, well-being, and work performance. Furthermore, in accordance with *Revista Brasileira de Medicina do Trabalho* guidelines, articles published more than five years ago were not included to ensure that current and relevant findings were analyzed.

Study selection was performed in two successive stages. First, the titles and abstracts were read to identify potential studies. In the second stage, the full texts of the selected articles were analyzed regarding the adequacy of the objectives, methodological consistency, and relevance of the results. As recommended in this journal’s guidelines, the following criteria were used to ensure the quality of the evidence: Strengthening the Reporting of Observational Studies in Epidemiology criteria were used for observational research, the Consolidated Standards of Reporting Trials for randomized clinical trials, and the Preferred Reporting Items for Systematic Reviews and Meta-Analyses for systematic reviews.

The data from the selected studies were synthesized and analyzed qualitatively. The main strategies, factors that helped or hindered their implementation, and the effects on worker health and well-being were identified. To ensure coherence and consistency, the results were presented in predetermined thematic categories based on the collected evidence. This allowed for connections between the different approaches and highlighted common elements of successful interventions. We also sought to contextualize the findings in Brazil, identifying the challenges inherent to adapting these strategies to the national market, in line with current public occupational health policies.

Finally, in compliance with the ethical principles of scientific research, all studies included in this review were duly cited to ensure its transparency and integrity. Furthermore, given the importance of open science for the dissemination of knowledge, all sources are available in open-access repositories, enabling replication of the procedures and deeper discussion.

## RESULTS AND DISCUSSION

The analysis indicated that strategies to promote health and well-being in the workplace are a set of integrated actions whose effects go beyond illness prevention, directly affecting quality of life, productivity, and sustainability. The results show that structured interventions, combined with an organizational culture focused on employee care, significantly reduce absenteeism and presenteeism and strengthen the bond between employees and companies, creating healthier and more motivating environments.^2,3^

Promoting well-being in the workplace transcends traditional occupational health practices by integrating the physical, mental, and social aspects of employee health. This reflects a holistic understanding of human beings, recognizing that psychological pressure, motivation, and a sense of belonging are fundamental to professional performance and personal satisfaction. A humanized approach that recognizes the intrinsic value of each individual not only promotes well-being but strengthens engagement, innovation, and resilience in organizations.

Regarding physical health strategies, a model called Workplace Health Promotion Embedded in Medical Surveillance stood out. Its effectiveness stemmed from integrating medical surveillance and health promotion. This model allows for early detection of occupational risks and preventive and educational actions tailored to the specific needs of each work environment.^2^ In addition to low cost and operational viability for companies of all sizes, this model has proven capable of improving worker quality of life while contributing to the sustainability of organizations, highlighting the strategic role of occupational medicine in building safer and more productive environments.

However, interventions focusing on psychological well-being demonstrated equally significant effects, highlighting the importance of mental health promotion as an essential element for emotional balance and workplace motivation. Policies and practices focused on emotional resilience, psychosocial support, and the development of socio-emotional skills has proven effective in reducing stress, improving the organizational climate and increasing employee satisfaction, thus strengthening team cohesion and retaining talent.^3,4^ Barry^8^ corroborated these findings, demonstrating that mental health promotion in the workplace reduces the effects of occupational stress, strengthens employee engagement, and improves professional performance, creating a virtuous cycle that benefits both workers and organizations.

An analysis of international experiences highlighted the importance of coordinating public policies and corporate initiatives to create healthier and more inclusive workplaces. In Hong Kong, the Joyful@Healthy Workplace program integrated educational campaigns, psychological support, and ergonomically appropriate work environments, significantly reducing absenteeism and presenteeism while increasing employee satisfaction and retention.^7^ The European Union’s OHS Strategy 2020 demonstrated that expanding access to quality health services reduces occupational risk and strengthens social inclusion and economic sustainability, highlighting the role of occupational health as a determining factor for human and organizational development.^6,10^

The organizational factors that influenced the effectiveness of these strategies were commitment by leadership, active employee participation, and the development of work environments based on respect and appreciation. According to Pescud et al.,^1^ employee engagement in occupational health initiatives depends largely on how these actions are communicated and implemented, highlighting the importance of a participatory and transparent approach that involves all organizational levels. An organizational culture that values employee well-being also proved crucial for overcoming cultural barriers and consolidating sustainable practices, highlighting the need for continuous integrated actions, rather than isolated interventions.^5,9^

Leadership plays a critical role in the development of an organizational culture focused on well-being. Managers who are committed to employee health inspire trust and create a supportive environment that encourages open communication and collaboration. Such active leadership facilitates the implementation of effective occupational health policies and encourages employees to actively participate in well-being programs, making each team member an engaged participant in promoting a healthier and more inclusive workplace.

In Brazil, challenges to implementing these strategies are largely related to cultural resistance, limited investment in occupational health, and difficulties measuring long-term results. Adapting international best practices to the national market requires an approach that considers the sociocultural characteristics of Brazilian workers and the structural characteristics of companies of different sizes and sectors. Building partnerships between the public and private sectors while developing more comprehensive and accessible public policies has proven essential for expanding worker access to quality occupational health services, reducing inequality and strengthening social inclusion.^5,6^

Mental health promotion in the workplace can help reduce the stigma of mental disorders, while offering appropriate support can transform the workplace into a space where individuals feel safe to seek help and express their emotions. Programs that foster emotional resilience and offer psychological support resources have been shown to significantly reduce stress and anxiety levels and improve individual well-being, in addition to collective effectiveness and team cohesion.

Integrating health surveillance and occupation illness prevention was one of the main factors in the success of the analyzed strategies, which highlights the importance of continuous monitoring of occupational risk in combination with programs to promote physical and mental well-being.^2,4^ The Total Worker Health model, which integrates health, safety and well-being, exemplified how a holistic approach can contribute to lower occupational risk and increased productivity, creating safer, healthier, and more motivating work environments.^5^ This perspective, which considers the worker as a whole, has proven particularly relevant in contexts of high psychological pressure and intense physical demands, highlighting the need for initiatives that address both disease prevention and the strengthen emotional and social well-being.

Public awareness campaigns about the importance of mental health in the workplace can help reduce stigma, encouraging individuals to seek support without fear of discrimination. Partnerships between the public and private sectors can catalyze innovation, enabling the creation of accessible technological solutions, such as health monitoring apps and platforms for remote psychological support.

Cross-sector collaboration is essential for developing guidelines that respect the cultural and economic characteristics of each region. This ensures that both large corporations and small businesses can implement successful practices. Furthermore, creating regulatory bodies to monitor and evaluate the effectiveness of these policies is vital to ensure continuous improvement and adaptability to changes in the labor market.

Adapting successful international practices to local needs is essential to ensuring their effectiveness in Brazil. This involves both financial investment and a deep understanding of the cultural and social dynamics that influence the workplace. By developing clear indicators to measure the effects of these practices, organizations can tangibly demonstrate the benefits of investing in occupational health, in terms of reduced medical costs and absenteeism, as well as productivity, job satisfaction, and team cohesion.

Finally, the success of strategies to promote health and well-being in the workplace depends on corporate ability to coordinate occupational illness prevention initiatives, psychosocial support, and the development of an organizational culture focused on employee care, combining these in a continuous and sustainable manner. The evidence of the included studies confirmed the hypothesis that integrated strategies contribute to reduced occupational risk, increased productivity, and improved organizational climate, providing benefits for both workers and companies. However, overcoming the challenges of cultural resistance, limited investment, and measurement of the results requires commitment by leadership, active participation by employees, and stronger public policies regarding occupational health, thus resulting in healthier, more inclusive, and sustainable workplaces.

## CONCLUSIONS

This review of strategies to promote health and well-being in the workplace showed that the effectiveness of these initiatives fundamentally depends on the integration of preventive actions and psychosocial support to develop an organizational culture that values physical, emotional, and social dimensions of employee care. Programs that combine medical surveillance with health promotion, such as Workplace Health Promotion Embedded in Medical Surveillance, have had positive effects on employee quality of life and company sustainability, highlighting the importance of approaches that go beyond disease prevention, seeking to strengthen the overall well-being of workers.

In this context, mental health promotion has emerged as an essential element in maintaining emotional balance and motivation in the workplace. It has been demonstrated that policies focused on emotional resilience and psychological support significantly reduce stress, strengthen team cohesion, and increase productivity. The Joyful@Healthy Workplace program developed in Hong Kong exemplifies the integration of educational campaigns, psychological support, and ergonomically appropriate work environments, significantly reducing absenteeism and presenteeism while increasing employee satisfaction, talent retention, and organizational climate.

International experiences, such as the European Union’s OHS Strategy 2020 and the Total Worker Health model, indicated that expanding access to quality occupational health services while creating safe and inclusive work environments reduces occupational risk and strengthens social inclusion and economic sustainability, consolidating the role of occupational health as a determining factor in human and organizational development. However, the effectiveness of these strategies is directly related to commitment from leadership and active participation from employees. Thus, continuous and integrated programs are needed, rather than isolated initiatives.

In Brazil, it will be challenging to adapt best practices to the sociocultural characteristics of the population and to different economic sectors, requiring investment in occupational health and the development of partnerships between the public and private sectors to expand worker access to quality health services and reduce inequality. It will be essential to measure the results of these initiatives to assess their effectiveness and long-term sustainability, which will require the development of monitoring indicators for their effects on health, productivity, and organizational climate.

Given the above, it can be concluded that integrated strategies based on a holistic approach that encompasses risk prevention, the promotion of physical and mental well-being, and psychosocial support are the most effective path to building healthier, more motivating, and more productive work environments. However, the success of these actions depends on the commitment of leadership, the active participation of employees, and the creation of an organizational culture based on respect and appreciation, consolidating a management model that, in addition to promoting corporate growth, contributes to social and economic development in a sustainable and equitable manner.
